# Objective measures of rollator user stability and device loading during different walking scenarios

**DOI:** 10.1371/journal.pone.0210960

**Published:** 2019-01-30

**Authors:** Eleonora Costamagna, Sibylle B. Thies, Laurence P. J. Kenney, David Howard, Ulrich Lindemann, Jochen Klenk, Rose Baker

**Affiliations:** 1 Centre for Health Sciences Research, School of Health & Society, Salford University, Salford, United Kingdom; 2 School of Computing, Science and Engineering, Salford University, Salford, United Kingdom; 3 Department of Geriatrics and Clinic for Geriatric Rehabilitation, Robert-Bosch- Hospital, Stuttgart, Germany; 4 School of Business, Salford University, Salford, United Kingdom; University of Indianapolis, UNITED STATES

## Abstract

Walking aids are widely used by older adults, however, alarmingly, their use has been linked to increased falls-risk, yet clinicians have no objective way of assessing user stability. This work aims to demonstrate the application of a novel methodology to investigate how the type of walking task, the amount of body weight supported by the device (i.e., device loading), and task performance strategy affect stability of rollator users. In this context, ten users performed six walking tasks with an instrumented rollator. The combined stability margin “SM” was calculated, which considers user and rollator as a combined system. A Friedman Test was used to investigate the effects of task on SM and a least-squares regression model was applied to investigate the relationship between device loading and SM. In addition, the effects of task performance strategy on SM were explored. As a result, it was found that: the minimum SM for straight line walking was higher than for more complex tasks (p<0.05); an increase in device loading was associated with an increase in SM (p<0.05); stepping up a kerb with at least 1 rollator wheel in ground contact at all times resulted in higher SM than lifting all four wheels simultaneously. Hence, we conclude that training should not be limited to straight line walking but should include various everyday tasks. Within person, SM informs on which tasks need practicing, and which strategy facilitates stability, thereby enabling person-specific guidance/training. The relevance of this work lies in an increase in walking aid users, and the costs arising from fall-related injuries.

## Introduction

Falls and fall-related injuries among older people are a major health problem; around 40% of the over 65s living at home are estimated to fall at least once a year, with around one in forty of the falls leading to hospitalisation [1]. The incidence of falls and the severity of the consequences increase rapidly with age [1, 2], cost the NHS an estimated £2.3 billion per year [3], and have major social impacts on the individual and their families [4]. As the number of over 65s is due to double by 2050, without changes to falls prevention strategies, the number of fall-related injuries is also likely to increase.

Regarding the circumstances of falls, ‘walking’ has been reported as the activity during which 48% of community-dwelling residents came to fall [5]. Walking is an activity during which a walking aid such as a walking stick or walking frame can provide weight-bearing support. Indeed, 22% of older adults in the UK use a walking aid indoors, and 44% use one outdoors [6]. However, rather counter-intuitively and the motivation for our research, general walking aid use (classified on a “yes”/”no” basis) has been shown to be a risk factor for falling [7], and injuries have been reported due to falling “whilst using” a walking aid [8]. Unfortunately, this published data fails to capture any detail on how the devices may have been used, in general or at the time of the fall. To date, walking aid use as a means of fall prevention remains an under-researched area. Without doubt users of walking aids are intrinsically vulnerable and therefore likely to fall. For a walking aid to be effective in preventing a fall, it first and foremost must be used in a stable and safe manner. However, at this time it is unknown whether walking aids are used according to the user guidance and training currently provided, and whether that guidance/training indeed facilitates stable, and therefore safe, use of walking aids.

Nevertheless, a vast number of clinical and manufacturer leaflets exist that aim to provide straight-forward instructions to users of walking aids. Guidance is generally brief, with varying levels of detail between clinical trusts and/or manufacturers. Basic instructions appear sensible, however, they fail to address everyday tasks such as turning in confined spaces, opening doors, and negotiating obstacles and changes in flooring level, some of which have previously been reported to be problematic [9]. Moreover, adherence to guidance is only judged via visual inspection, and the value of this is doubtful since “good use” is based entirely on subjective observation, often for only a small number of steps taken in a straight line. To date, guidance has not been validated in relation to user stability when performing a range of everyday walking tasks, and this is of concern, especially considering the rise in walking aid users within the ageing population [6].

In this context, we recently developed a novel, objective measure of the stability of walking aid users [10]. The measure, termed the combined (or system) stability margin (SM_System_), is calculated using force measurements taken from each of the walking aid legs and the user’s shoes, together with the position of the anatomical feet relative to the walking aid. The smaller the SM, the closer the system is to the point of tipping over, and the more susceptible it is to perturbations. To measure the required forces for calculation of SM, we initially instrumented a walking frame without wheels (“pick-up walker”) with load cells, which together with pressure sensing insoles and optical motion tracking allow for calculation of SM. Our approach is novel in that it considers the person and their walking aid to be a single system. We proved that combining information of walking aid and person is vital to accurately evaluate overall stability [10], and our approach is generalizable to a range of walking aids, including wheeled rollator frames (‘rollators’). Rollators are of particular interest because their general use has been shown to be ineffective in terms of preventing serious fall-related injuries [11]. Interestingly, and of direct relevance to this work, users of rollators have complained about lack of training [12], with as many as 81% having received no instructions or training at all regarding the use of their device [13].

Our approach, which provides an objective measure of stability, has the potential to support current clinical practice through evidence-based training. In the work reported here, we adapted our approach for use with a 4-wheeled rollator frame and, in a cohort of 10 in-patients in a geriatric ward, assessed stability across a range of everyday tasks. The objectives were:

1. Investigate the effects of the task being undertaken on stability. *Hypothesis*: Stability is greatest for straight line walking as compared to more complex tasks such as turning or obstacle crossing.
2. Investigate the relationship between device loading (i.e. the amount of body weight supported by the rollator) and stability. *Hypothesis*: Increased leaning onto the rollator causes the centre of pressure of the system to move forward into the base of support, thereby increasing stability.
3. Investigate the effects of rollator use strategy on stability: *Hypothesis*: the strategy employed for performance of a single task may either facilitate or impede stability.

## Materials and methods

### Quantification of stability

**Fig 1:**
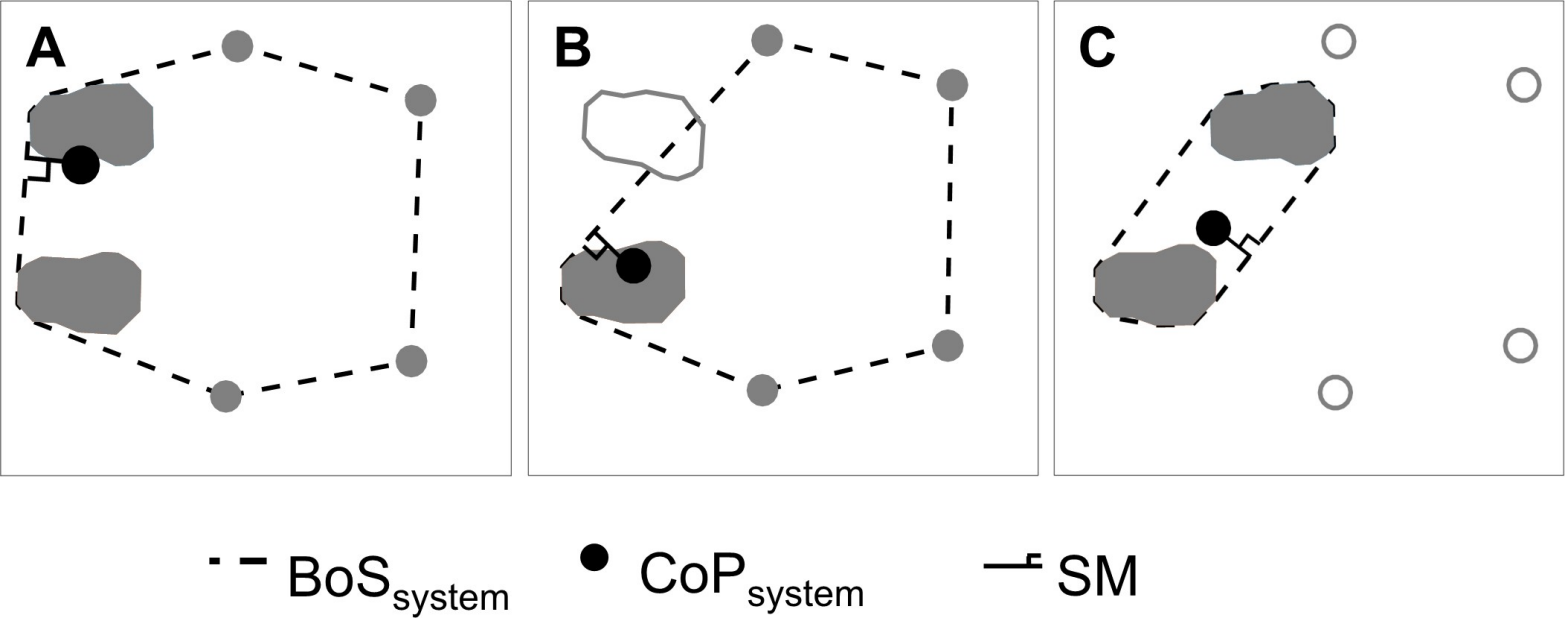
Examples of combined centre of pressure, combined base of support and system Stability Margin. Examples of combined centre of pressure, combined base of support and system Stability Margin for 3 cases: A) all 6 feet on the ground; B) 4 rollator feet on the ground and user in single support on their right foot; C) user in double support and rollator fully airborne (e.g. being lifted up a step). Grey foot prints indicate feet that are grounded; white foot prints indicate feet that are airborne.

Stability of rollator users was quantified using the methodology developed by Costamagna et al. [10], which, for the first time, looked at the user and their walking aid as a single combined system and quantified stability by calculating the combined (or system) Stability Margin (SM_System_) of user and device from wheel-force, insole-pressure, and position data using bespoke Matlab algorithms. SM_System_ is defined as the shortest distance between the Centre of Pressure of the combined system (CoP_System_) and the nearest edge of the combined Base of Support (BoS_System_) (Fig 1) and indicates how far the system is from tipping; hence, the higher SM_System_, the more stable the system is. Fig 1 shows how the size of BoS_System_ can vary, including double support with the rollator grounded, single support with the rollator grounded, and double support with the rollator lifted (e.g. when the user is in the process of stepping onto a kerb). It seems reasonable to think that when BoS_System_ is smaller, SM_System_ is also likely to be smaller; but this should not be confused with an unsafe gait as BoS_System_ may well be being sensibly utilised. For this reason, SM_System_ has been normalised by a parameter representative of the size of BoS_System_ (Eq 1).

$$SM=\frac{{SM}_{System}}{\sqrt{{Area(BoS}_{system})}}$$ Eq 1

The normalised SM_System_ (referred to simply as SM in this work) is dimensionless as it is the ratio of two lengths.

Finally, to further characterise rollator use, we also present the movement pattern (foot placements in relation to rollator movements), and device loading (DL), defined as the percentage of body weight transferred to the device through the user’s upper limbs.

### Instrumentation

**Fig 2:**
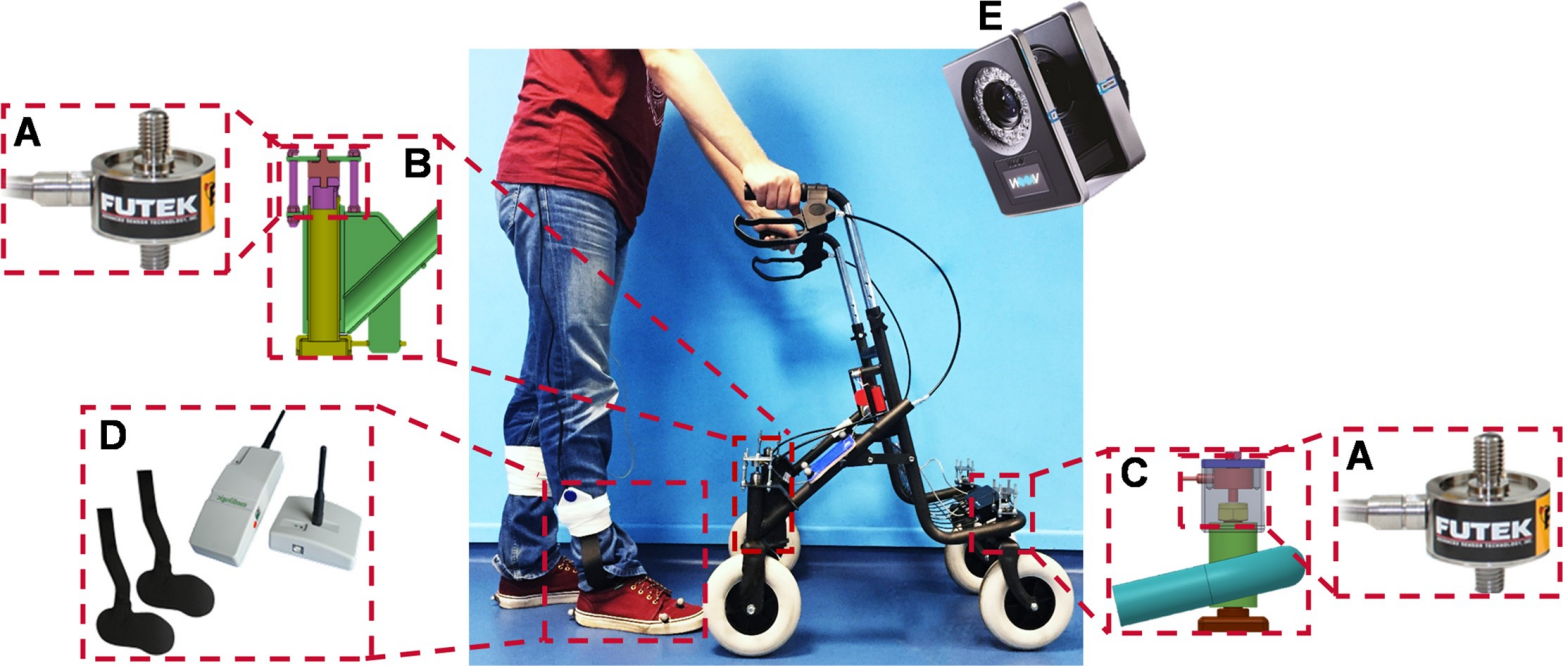
Instrumented rollator system. Details of: A) load cell; B) rear leg design; C) front leg design; D) pressure-sensing insole system; E) infrared camera.

For the purpose of this study, the technology of our original instrumented pick-up walker [1] was adapted for a 4-wheeled rollator. The instrumentation includes 4 single axis load cells (Futek LCM300, FUTEK Advanced Sensor Technology Inc., Irvine, California) and corresponding transmitters (Mantracourt T24-ACMi, Mantracourt Electronics Ltd., Exeter, UK), a pressure-sensing insole system (Medilogic insole, T&T Medilogic Medizintechnik GmbH, Schönefeld, Germany), and an 8-camera motion capture system (Vicon, Oxford, UK). Since the geometry and the characteristics of a rollator differ significantly from that of a pick-up walker, device-specific design modifications were necessary to integrate the load cells into the rollator legs in such a way that they accurately measure the vertical walker ground reaction forces; Fig 2 illustrates the modified rollator design.

The accuracy of CoP_System_ as derived from the load cell and insole data was tested by placing the device onto a force plate (600 × 900 mm AMTI BP600900) and asking a user to step onto the same force plate while holding the rollator. Subsequently, CoP_System_ was calculated from “gold standard” force plate data and compared to CoP_System_ as calculated from instrumented rollator data. Results of this accuracy evaluation are shown in S1 File.

### Participants

Ten rollator users aged 84.2 ± 5 years were recruited from the Geriatric Rehabilitation Clinic of the Robert-Bosch-Hospital Stuttgart, Germany. Inclusion criteria were: 1) age 65 years or older, 2) able to walk household distances with a rollator, but not able to walk such distances unaided. Exclusion criteria were: 1) history of head injury or concussion, 2) visual disorders not correctable by glasses, 3) diagnosed peripheral or central nerve dysfunction, 4) terminal disease, 5) inability to follow verbal instructions. Of the 10 participants, 8 were women, and 5 had a previous fall-related history of lower limb fracture. Moreover, 4 participants were new users, 2 participants had had their rollator for less than 6 months, and the remaining 4 participants were experienced users (had used the rollator for more than 6 months). Additional descriptive parameters are presented in Table 1. Comparative tests to investigate differences among participants on the descriptive parameters presented have been conducted, and results are reported in Supplement 2. Written informed consent was obtained from all participants, and the experimental protocol was approved by the University of Tuebingen Medical Faculty Ethics committee (678/2016BO1) and the University of Salford Ethics Committee (HSCR13-48).

**Table 1:** Descriptive parameters of study participants.

	Age (years)	SEX (M/F)	weight (kg)	height (cm)	BMI	FCI	FRACTURE	gait speed (m/s)	Rollator use (months)
**P1**	87	F	78	154	32.9	3	no	0.61	<0.5
**P2**	78	F	60	153	25.6	5	yes	0.65	0.5–6
**P3**	85	F	54	152	23.4	3	no	0.65	0.5–6
**P4**	83	F	71	160	27.7	4	no	0.51	<0.5
**P5**	79	M	71.5	168	25.3	6	yes	0.76	<0.5
**P6**	91	F	47	154	19.8	5	yes	0.48	>6
**P7**	82	F	60	153	25.6	3	no	0.37	>6
**P8**	77	F	80	161	30.8	4	yes	0.27	<0.5
**P9**	91	F	55	154	23.2	7	yes	0.71	>6
**P10**	89	M	79	158	31.6	3	no	0.50	>6

BMI: Body Mass Index; FCI: Functional Comorbidity Index (number of diseases and symptoms, from a maximum of 18)

### Protocol

The experiments took place in the gait laboratory of the Robert-Bosch-Hospital in Stuttgart, Germany. All participants performed 6 tasks with the instrumented rollator representative of activities of daily living: straight line walk (5 m); 90° turn; 180° turn; obstacle crossing (involving pushing two wheels of the rollator over the end part of a long wooden beam, cross section 22 mm high and 62 mm wide, while the other two wheels remain on the level floor); backwards walk (2.5 m) as if to open a door; and negotiating a 50mm step up. Participants performed each task twice at their self-selected speed. As it was expected that some participants might experience fatigue during the assessment, the order in which tasks 2–6 were performed was rotated.

### Data analysis

#### Effects of task on stability margin

To test whether the task has an effect on stability and, if so, which tasks are more or less challenging to the user’s stability, the minimum SM for each task (which corresponds to the instant during the whole task in which the user was the least stable) was compared to the minimum SM of all other tasks including straight line walking. For this, a Friedman test, which is robust to non-normality, followed by post-hoc one-sided Wilcoxon Signed-Rank Tests was run in R [14] to analyse the effects of task on stability. Finally, to account for the fact that multiple conditions were tested in this study, all p-values were adjusted using a Bonferroni correction.

#### Relationship between stability margin and device loading

The relationship between SM and DL was investigated with a least-squares regression for each participant and for each task using a purpose-written FORTRAN program:

$${SM}_{ij}=\alpha_{ij}+\beta_{ij}{DL}_{ij}$$

Where i = 1,2,…,10 indicates the i^th^ participant, j = 1,2,…, 6 the j^th^ task, and α and β are, respectively, the intercept and slope of the model.

However, since SM and DL are both time series, the assumption that observations are independent could not be made; therefore, the time series have been first differenced obtaining ΔSM_ij_ and ΔDL_ij_, and then, since ΔSM_ij_ and ΔDL_ij_ showed very low autocorrelation (<0.1), the least-squares regression without intercept was calculated (Δ*SM_ij_* = *β_ij_*Δ*DL_ij_*). Finally, the overall regression coefficient, β, was obtained by calculating the weighted mean of all the β_ij_ with weights given by the computed inverse variance of β_ij_.

To explore whether regression coefficients vary statistically between participants and tasks, a general linear model computed in SPSS was used, with tasks and patients as fixed effects.

#### Effects of step-up strategy on stability

To explore the effects of different strategies on stability, the strategy used spontaneously by each participant to get up the 50mm step was recorded, together with the corresponding minimum SM. How the adopted strategy influenced the corresponding minimum SM was then examined.

## Results

All tasks were completed by all patients within a maximum of 20 minutes and without any obvious signs of fatigue (not tested).

### Illustrative data on rollator use and user’s stability

**Fig 3:**
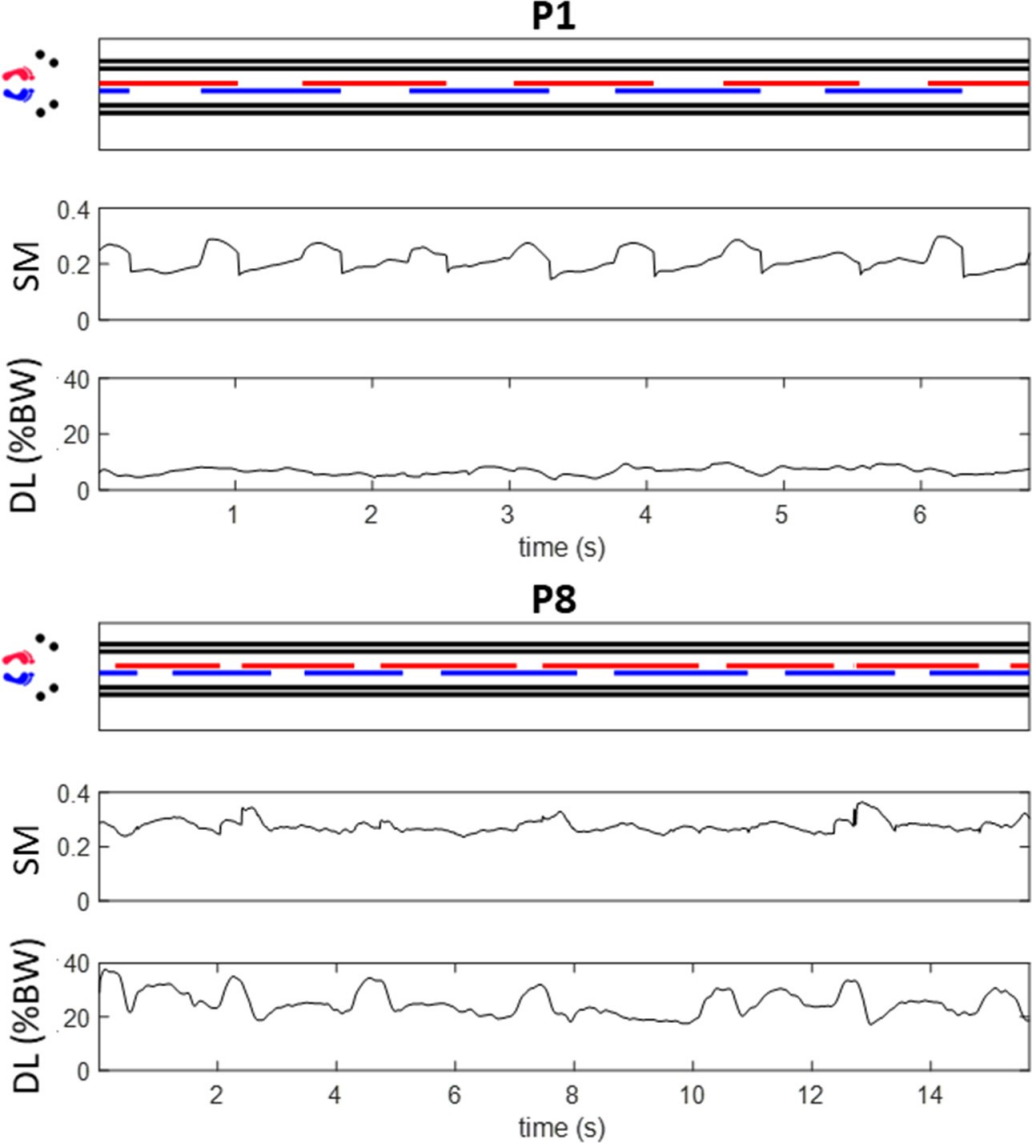
Example data sets for two rollator users (P1: Fit, P8: Frail) walking in a straight line. Top: movement pattern showing times when wheels and feet are in contact with the ground. Middle: stability margin ‘SM’. Bottom: device loading ‘DL’.

To illustrate the measures derived from our instrumented rollator system, Fig 3 shows the movement pattern, i.e. the times when wheels and feet are in contact with the ground, together with SM and DL data for straight line walking of two rollator users (P1 and P8). P8 was identified by the research team as a relatively frail user, who was limping due to history of hip fracture (right hip) and because he had the lowest gait speed (0.27 m/s) [15], whilst P1 was deemed fittest based on gait speed and functional comorbidity index. Surprisingly, it can be observed from the graphs that P8 has a generally higher SM (SM = 0.24–0.36) than P1 (SM = 0.15–0.30). However, P8 also shows a much greater DL (up to 37% BW) than P1 (up to 10% BW), and this may affect the observed absolute values of SM. It is further notable that P1’s DL remains approximately constant throughout the walking trial with a mean value of 6.8 ± 1.3% BW, whilst P8’s DL is less regular, showing greater variability (25.1 ± 4.6% BW). Last but not least, it can be observed that DL considerably increases every time P8 is swinging the left foot forward, indicating a need for additional support from the rollator when standing in single support on the limb that was previously fractured.

### Effects of task on stability

**Fig 4:**
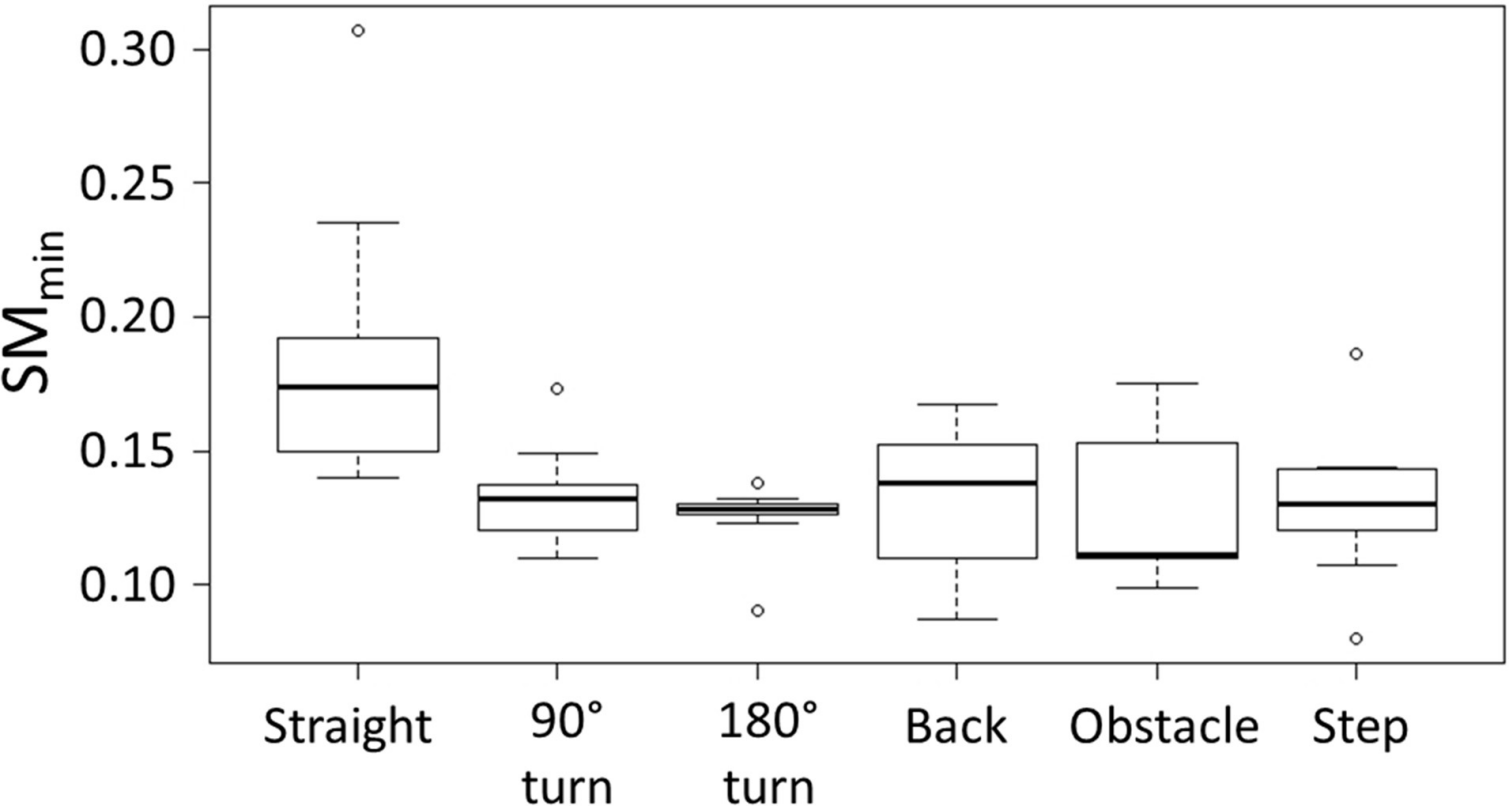
Box plot of the minimum SM for each task across the 10 participants. The bottom and top edge of the boxes indicate the first and third quartile, the thicker line inside the box represents the median, and the whiskers below and above the box show the minimum and maximum values respectively. Circles denote outliers as identified automatically by the software R. “Straight”: straight line walking, “Back”: Backwards walking, “Obstacle”: obstacle crossing, and “Step”: stepping up a kerb.

Fig 4 presents the distribution of the minimum SM for each task across the 10 participants, showing a visible difference between straight line walking and all other tasks. Corresponding results of the Friedman test showed that the task performed has a significant effect on stability (χ^2^ = 20.2, degrees of freedom = 5, p = 0.001), and the post-hoc Wilcoxon tests used to further investigate the effect of the individual tasks confirmed that that the minimum SM for each task is significantly lower than that during straight line walking (Table 2). Furthermore, the effect size |r| > 0.5 indicates that each task has a large effect on stability. Regarding the comparison between all other tasks, however, no further significant differences between the more complex tasks emerged from the Wilcoxon tests.

**Table 2:** Group median values of SM_min_ for each task and results of the Wilcoxon Signed-Rank Test. “Straight”: Straight line walking, “Back”: Backwards walking, “Obstacle”: Obstacle crossing, and “Step”: Stepping up a kerb.

	median (mm)	z-score	p value	effect size R
**Straight**	0.1665			
**90° turn**	0.1260	-2.8067	0.006	-0.8876
**180° turn**	0.1275	-2.8031	0.012	-0.8864
**Back**	0.1380	-2.6679	0.006	-0.8893
**Obstacle**	0.12	-2.8031	0.006	-0.8864
**Step**	0.1280	-2.8049	0.006	-0.8870

### Relationship between stability margin and device loading

**Fig 5:**
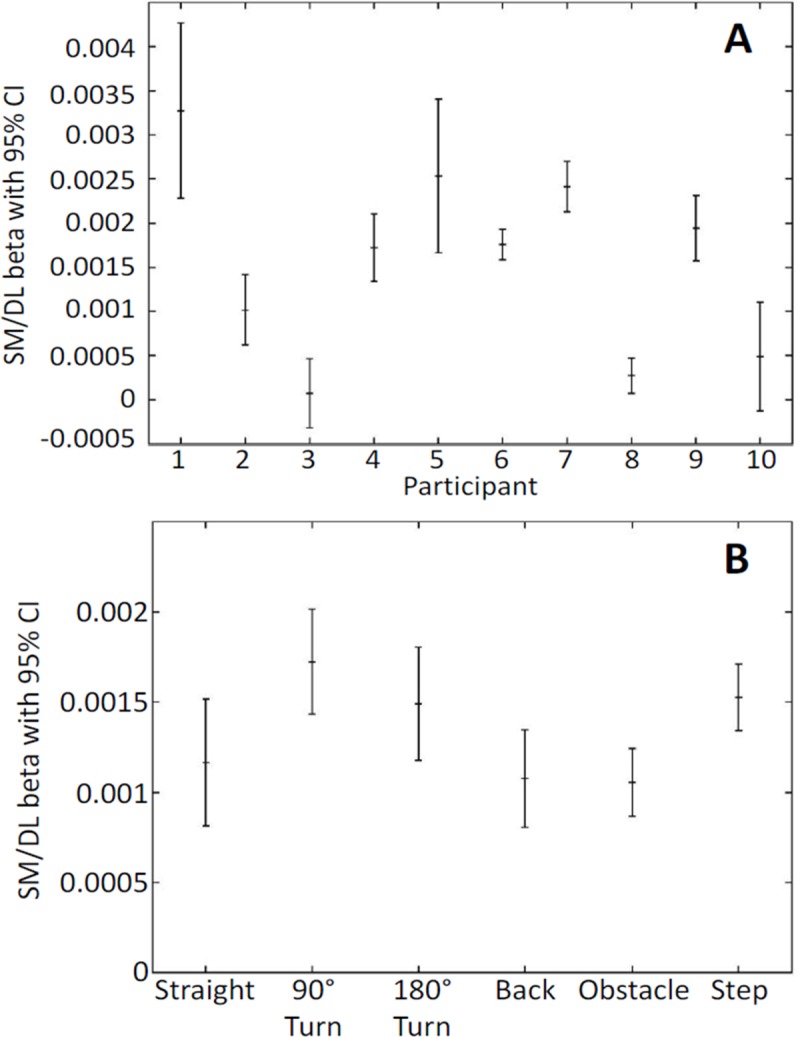
Mean (weighted) values of β for A) the 10 participants and B) the 6 tasks.

Results of the least-squares regression confirmed that the overall weighted regression coefficient, β, is significantly positive (β = 0.000695 ±0.000036, z-score = 18.51) and, hence, that SM increases with DL. Moreover, the general linear model showed that β varies significantly between participants (p<0.001) but not between tasks (p = 0.69). The mean (weighted) values of β for the 6 tasks and the 10 participants are shown in Fig 5.

### Effects of strategy used on stability margin

Three different strategies to step onto a 50 mm-high platform were observed in the rollator users tested. Seven out of ten users adopted a lateral approach which consisted of:

- User lifts either the right or left side of the rollator first;
- User places the front wheel of the lifted side onto the platform;
- User lifts the rest of the rollator and places it onto the platform.

Two users adopted an “all together” approach (referred to as “All together V1”), using the handles to lift the rollator up completely in a single manoeuvre and placing all 4 wheels simultaneously on top of the platform. Finally, 1 user adopted an alternative “all together” approach (referred to as “All together V2”) as follows:

- User leans forward and grabs the rollator horizontal bar with one hand while holding one handle with the other hand;
- User lifts the rollator up completely in a single manoeuvre.

Of the 3 different approaches adopted by the rollator users, the lateral approach was the one with the highest minimum SM (0.13 ± 0.03), followed by the “All together V1” approach (0.12 ± 0.01) and, lowest of all, the “All together V2” approach (0.11; since only 1 trial is available for this approach, standard deviation could not be calculated).

## Discussion

This is the first study that, in a cohort of 10 in-patients in a geriatric ward, assessed stability with an instrumented rollator for six everyday walking tasks. Stability was investigated with an objective assessment methodology previously developed by the authors [10], which treats the user and their rollator as a single combined system and informs on the corresponding stability margin, i.e. how far the system is from the point of “tipping over”. It is noteworthy that since its initial development [10], the method has been further refined in that SM_System_ is now normalized to take into account the size of the BoS, because a smaller SM_System_ may simply be the consequence of a smaller BoS. However, despite this normalization, the sample data of two participants (one fit and one comparatively frail) indicate that, whilst SM is a direct measure of stability (nearness to tipping), a larger SM (as observed for the more frail user) does not necessarily mean a safer gait and lower risk of falling. For instance, those users who need a rollator only as a balance aid, and which do not transfer substantial amounts of body weight onto the device, will likely have a lower SM than those who need it for weight-bearing support. In future, a measure representative of overall stability that does not depend on absolute values of SM would be more informative, especially when it is the aim to characterize relative stability of participants.

Investigating effects of task on SM, the data provide evidence that stability decreases substantially from straight line walking to all other tasks. Yet, no significant differences between the other tasks (90° turn, 180° turn, backwards walk, obstacle crossing, and step up/down) could be found, indicating that the level of challenge for each task may be similar or subjective. These findings provide initial evidence as to how safe use of a rollator may be facilitated: training should target daily activities other than straight line walking, and it should be customised to focus on those activities for which a user exhibits particularly low stability.

Investigating stability in relation to device loading, findings showed that SM generally increases significantly when DL increases, thereby supporting our hypothesis that as more weight is transferred onto the rollator, CoP_System_ moves forward towards the centre and away from the edges of BoS_System_. Hence, advice to those who show generally low SM, and especially to those whose SM is often directed towards the rear of BoS_System_, could include instruction to lean more onto the rollator. However, we acknowledge that this may not always be possible if users have weak upper limbs, and it may also lead to new pathologies such as tendonitis or osteoarthritis [16, 17]. Furthermore, it must be considered that prolonged offloading of the lower limbs may induce lower limb weakness.

It must also be noted that, considering the different strategies used to stepping up a kerb, it may be that the relationship between SM and DL is influenced by the specific strategy used and may need to be treated separately from other tasks. Future work needs to investigate this further, however, at this time the authors did verify in a post-hoc analysis that the exclusion of the stepping-up task from the regression and general linear models did not change the significance of the models and neither the results for the remaining tasks.

Finally, for the first time, we have shown how stability data can inform rollator use strategies. Specifically, when going up a step the “lateral approach” appears to be the most stable strategy, because the rollator never leaves the ground completely and hence provides the user with continued support; the “All together” approaches, instead, require greater strength as the user has to lift the rollator in one manoeuvre and leave him/her with no support during the lifting-up phase. The authors note that none of the participants went up the step as stated in some guidance documents, i.e. lifting the front wheels first, then the rear, then squeezing the brakes before stepping up.

One limitation that emerges from the above discussion is that SM, despite its normalization by ($\sqrt{area(BoS)}$), appears to be further influenced by other factors. For example, it was observed that P8 (user with history of hip fracture and with a gait speed of 0.27m/s) presented values of SM higher than the user who walked more than twice as fast and had no history of hip fracture (P1); this is likely due to SM being influenced by user-specific factors such as the amount of body weight transferred onto the rollator. Hence, we conclude that the stability margin alone provides useful information within user (e.g., in relation to which tasks need to be practiced and trained most and which task performance strategy is safer), but that, in order to compare users and inform on who’s at greater risk of falling, further work is needed. Therefore, future analysis should investigate additional factors such as DL and their effects on SM, and/or focus on analysis techniques that do not depend on absolute values of SM, for example, the regularity of the SM time series (e.g. as measured by autocorrelation) [18]. Indeed, we observed that the stability margin of the fitter user shows greater regularity than that of the frail user.

For these reasons, future work should investigate techniques such as autocorrelation functions applied to the stability margin to identify whether this approach may complement the methods presented here.

Finally, we acknowledge that the number of users tested in this work (10) is small, and that we have not validated SM against existing stability measures. Regarding the latter, however, to date, no gold standard measure adequate for assessment of stability of walking aid users exists: methods previously designed for unassisted walking such as investigations of linear variability of basic gait parameters [19] ignore that user and device are mechanically coupled and also assume periodicity of gait, whilst others [20] assume that gait can be described using inverted pendulum models and, hence, cannot be applied either. Therefore, longer-term, a prospective study on falls is needed to validate SM and generalise findings.

## Conclusions

In summary, our approach provides objective data on walking stability of the combined system (user and rollator) for a range of everyday tasks, and provides first insights into how to objectively assess alternative rollator use strategies to inform evidence-based training. The relevance of our approach lies in an increase in users of walking aids within our ageing population, and the associated costs arising from fall-related injuries. One key insight gained is that training should not be limited to straight line walking alone, but should include more complex tasks representative of daily walking activities. Within person, the stability margin SM can be used to identify which tasks needs to be practiced, and which strategy facilitates stable performance of a given task. Indeed, in principle, the use of instrumented rollators as assessment tools in clinics could enable person specific guidance and training. Longer-term, evidence-based training should increase the benefits of using rollators as a means of fall prevention.

## Supporting information

S1 File Validation of the instrumented rollator. (DOCX)

S2 File Participants’ descriptive parameters. (DOCX)
