# Supplementary material for: Objective measures of rollator user stability and device loading during different walking scenarios
Source: PLoS One. 2019 Jan 30;14(1):e0210960. doi: 10.1371/journal.pone.0210960 (PMC6353162; doi:10.1371/journal.pone.0210960)
Supplement: S1 File — (DOCX) [file pone.0210960.s001.docx]

**S1 File: Validation of the instrumented rollator**

A Root Mean Square difference equal to 10.18 mm in AP direction and 11.93 mm in ML direction were obtained when comparing the combined centre of pressure as calculated by the instrumented rollator to the corresponding data recorded with the force plate. With regard to the maximum difference, this was equal to 24.83 mm in AP direction and 19.8 mm in ML direction, which we considered acceptable being equal, respectively, to 3.67% and 3.36% of the maximum width and length of the combined BoS in the testing conditions studied. An example of the comparison between the combined CoP calculated by the instrumented rollator and that calculated by the force plate is shown in S1 Fig.


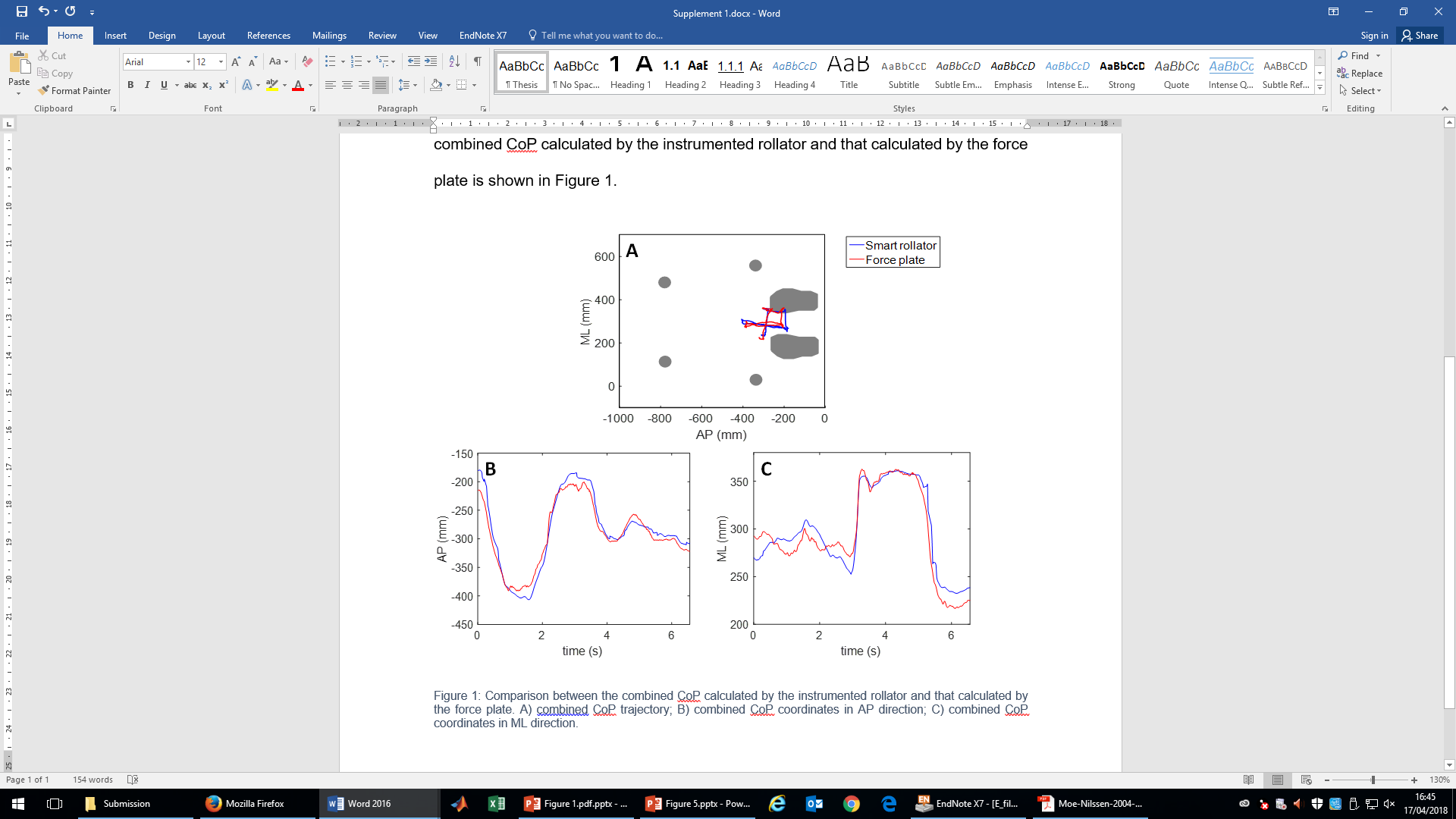


**S1 Fig**: **Comparison between the combined CoP calculated by the instrumented rollator and that calculated by the force plate.** A) combined CoP trajectory; B) combined CoP coordinates in AP direction; C) combined CoP coordinates in ML direction.
