# Supplementary material for: Objective measures of rollator user stability and device loading during different walking scenarios
Source: PLoS One. 2019 Jan 30;14(1):e0210960. doi: 10.1371/journal.pone.0210960 (PMC6353162; doi:10.1371/journal.pone.0210960)
Supplement: S2 File — (DOCX) [file pone.0210960.s002.docx]

**S2 File: Participants’ descriptive parameters**

Basic comparative tests to investigate differences among participants on the descriptive parameters presented in Table 1 have been conducted, and results are reported below.

The correlation between the following descriptive parameters has been tested using the Spearman’s correlation coefficient: gait speed and length of time using a rollator; gait speed and history of lower limb fracture; gait speed and Functional Comorbidity coefficient (FCI); gait speed and BMI; history of lower limb fracture and BMI; and history of lower limb fracture and FCI.

Of these, history of lower limb fracture and FCI were the only variables that were correlated (r = 0.87) and the difference in FCI between those with and without history of lower limb fracture was significant (p = 0.016) when tested with a Wilcoxon rank sum test.

However, correlation results must be interpreted considering the very small size of the sample.
